# Developing Resident-Sensitive Quality Measures for Internal Medicine

**DOI:** 10.1001/jamanetworkopen.2026.11700

**Published:** 2026-05-08

**Authors:** Brandon Tang, Andrew C. L. Lam, Matthew Wankiewicz, Marwa F. Ismail, Surain B. Roberts, Chang Liu, Daniel Schumacher, Benjamin Kinnear, Yoon Soo Park, Anjala Tess, Amol A. Verma, Fahad Razak, Martin Pusic, Shiphra Ginsburg, Brian M. Wong

**Affiliations:** 1 Division of General Internal Medicine, Department of Medicine, University of Toronto, Toronto, Ontario, Canada; 2 Li Ka Shing Knowledge Institute, Unity Health Toronto, Toronto, Ontario, Canada; 3 Division of General Internal Medicine, Department of Medicine, Unity Health Toronto, Toronto, Ontario, Canada; 4 Department of Medicine, University of Toronto, Toronto, Ontario, Canada; 5 Institute of Health Policy, Management and Evaluation, University of Toronto, Toronto, Ontario, Canada; 6 Department of Pediatrics, University of Cincinnati College of Medicine, Cincinnati, Ohio; 7 Division of Emergency Medicine, Cincinnati Children’s Hospital Medical Center, Cincinnati, Ohio; 8 Division of Hospital Medicine, Cincinnati Children’s Hospital Medical Center, University of Cincinnati College of Medicine, Cincinnati, Ohio; 9 Department of Medical Education, University of Illinois Chicago; 10 Department of Medicine, Harvard Medical School, Boston, Massachusetts; 11 Department of Laboratory Medicine and Pathobiology, University of Toronto, Toronto, Ontario, Canada; 12 Department of Pediatrics, Harvard Medical School, Boston, Massachusetts; 13 Research and Education Foundation, American Board of Medical Specialties, Chicago, Illinois; 14 Division of Respirology, Department of Medicine, Mount Sinai Hospital, Toronto, Ontario, Canada; 15 Centre for Quality Improvement and Patient Safety, Temerty Faculty of Medicine, University of Toronto, Toronto, Ontario, Canada; 16 Division of General Internal Medicine, Sunnybrook Health Sciences Centre, Toronto, Ontario, Canada

## Abstract

**Importance:**

The ultimate goal of residency education is to train physicians to deliver high-quality patient care. However, residents rarely receive data-driven feedback because resident-level quality measures are lacking.

**Objective:**

To develop and evaluate resident-sensitive quality measures (RSQMs) using electronic health record data to inform graduate medical education.

**Design, Setting, and Participants:**

This cohort study used call schedules linking senior internal medicine residents to patient admissions during overnight internal medicine call shifts at 5 teaching hospitals in Canada from July 1, 2010, through December 31, 2019. Using clinical practice guidelines, 7 RSQMs related to pneumonia or general care for all internal medicine admissions were developed. To support interpretation, a care variation framework was applied that categorized measures as low value (not recommended), discretionary (context dependent), or evidence based (recommended) to enable comparisons between observed and expected variation. The data were analyzed between March 1, 2024, and February 23, 2026.

**Main Outcomes and Measures:**

The low-value care RSQM measured potentially inappropriate red cell transfusions (all admissions). Discretionary RSQMs measured use of antibiotics, imaging, or blood work for either pneumonia or all admissions. The evidence-based care RSQM measured ordering of first-line antibiotics for pneumonia. Resident-level variation was assessed using descriptive statistics, including the median proportion of eligible admissions with each RSQM performed and interquartile range.

**Results:**

The cohort included 132 291 patient admissions (median [IQR] age, 70 [55-83] years; 50.6% male) linked to 793 residents. Residents had a median (IQR) of 187 (89-228) admissions, including a median (IQR) of 18 (10-24) admissions for pneumonia. Potentially inappropriate red cell transfusions occurred in a low proportion of encounters, with little variation (median, 0%; IQR, 0%-0%). Discretionary measures, including use of second-line antibiotics, advanced imaging, chest computed tomography, and serum protein electrophoresis, varied across residents. For pneumonia admissions (n = 13 470), the RSQM for first-line antibiotic use in pneumonia was sensitive to the time windows for included orders, ranging from 22% (3027 admissions) to 76% (10 205 admissions), depending on the cutoffs applied.

**Conclusions and Relevance:**

This cohort study outlined an approach to developing and evaluating RSQMs using readily available electronic health record data to evaluate internal medicine residents’ quality of care. Although the RSQMs showed potential, their use for inpatient internal medicine may be more appropriate at the program level due to unresolved concerns regarding attribution and statistical reliability.

## Key Points

**Question:**

Can electronic health record data be used to develop resident-sensitive quality measures (RSQMs) that provide physicians-in-training feedback on their quality of care?

**Findings:**

This cohort study of 793 senior internal medicine residents linked to 132 291 patient admissions found both expected and unexpected patterns of resident-level variation within a framework evaluating low-value, discretionary, and evidence-based care RSQMs. In particular, rates of first-line antibiotic ordering for pneumonia varied widely depending on the attribution time window.

**Meaning:**

These findings highlight challenges of attribution and statistical reliability that should be considered when developing and evaluating RSQMs using readily available electronic health record data.

## Teaser

This cohort study evaluates the outcomes of resident-sensitive quality measures developed using electronic health record data to inform graduate medical education in Canada.

## Introduction

The ultimate goal of residency education is to train physicians to deliver high-quality patient care. To support this objective, national accrediting bodies in Canada, the US, and internationally have invested heavily in competency-based medical education, which emphasizes achievement of patient-centered outcomes during training.^1^ Despite these efforts, assessment in competency-based medical education largely relies on direct observation of performance, which has known limitations, including rater subjectivity and inconsistent opportunities for observation.^2,3,4^ Given a lack of resident-level quality measures, residents rarely receive feedback on the quality of care they provide.^5^

To address this gap, Schumacher et al^5^ introduced the concept of resident-sensitive quality measures (RSQMs), defined as measures that are (1) important for patient care and (2) meaningfully attributable to individual residents. Resident-sensitive quality measures focus on clinical process measures that are more directly influenced by resident care decisions rather than downstream outcomes (eg, mortality), which are less directly affected by residents. Prior studies of RSQMs have found practice variation for common conditions in the pediatric emergency department (ED). However, these studies relied on manual medical record review for data extraction, limiting scalability and integration into residency training.^5^

Recent advances in data science may enable large-scale measurement of physician quality of care. For example, attending internal medicine physicians in Ontario, Canada, receive individualized quality-of-care reports through a province-wide quality improvement initiative.^6,7,8^ This measurement is facilitated by the General Medicine Inpatient Initiative (GEMINI) program, which aggregates electronic health record (EHR) data from more than 35 hospitals, representing more than 60% of the medical beds in Ontario.^9^ The GEMINI program combines detailed clinical data (laboratory, radiology, medication, and treatment orders) with administrative data (patient demographics, discharge diagnoses, and resource use) with 98% to 100% accuracy of selected data elements compared with manual medical record review.^10,11^ A recent review indicated growing interest in harnessing similar EHR data to support graduate medical education (GME) in the US and internationally.^12^

Providing residents with data-driven feedback on their quality of care could represent an important step toward integrating patient-centered outcomes into GME. This study thus aimed to develop and evaluate RSQMs using EHR data to inform GME.

## Methods

### Study Setting and Design

This cohort study examined senior (postgraduate year ≥2) internal medicine residents at the University of Toronto during overnight general internal medicine call shifts when they lead the inpatient medical teams. These shifts occurred across 5 urban teaching hospitals in Toronto. Patient admissions and select care orders from 6 pm until 8 am the following day (attribution time window) were attributed to the senior internal medicine resident on call. Our rationale for time-based attribution was based on our training program structure and practices, in which the senior resident triages all internal medicine admissions overnight, supervises junior trainees, and formulates treatment plans. Thus, the majority of clinical decisions during this period are directed by senior residents and discussed for the first time with an attending physician the next morning after call.

This study received Research Ethics Board approval from the University of Toronto, which exempted the need for informed consent due to the use of deidentified, aggregate data. The study followed the Strengthening the Reporting of Observational Studies in Epidemiology (STROBE) reporting guideline for cohort studies.

### Development of RSQMs

Our research team proposed candidate RSQMs through a consensus process guided by clinical practice guidelines.^5,13^ To inform interpretation, we drew on Wennberg’s existing framework to describe different variation patterns observed in health care.^14^ These patterns include variation due to underuse of effective care (evidence-based care), variation in relation to preference-sensitive care (driven by clinician and patient values), and variation in context of supply-sensitive care (driven by resources, eg, imaging availability).^14^ This framework informed our development of a novel care variation framework (CVF) that supported conceptually grounded comparisons between observed and expected variation across 3 categories: low value, discretionary, and evidence based. Low-value care processes lack evidence for a given clinical scenario and should usually be avoided; the expected pattern would be infrequent performance, with narrow variation. Discretionary care processes could vary based on the clinical context and clinician judgment; the expected pattern would be variable frequency and practice variation, depending on the specific care process. Evidence-based care processes should be performed according to clinical guidelines; the expected pattern would be frequent performance with narrow variation.

We also incorporated positive control measures, which represent routine care processes that are not resident attributable, to validate the accuracy and completeness of our data.^15,16^ For example, given that chest radiography is typically used to diagnose pneumonia, we included it as a positive control; a low-order proportion would raise concerns about data accuracy.

We proposed both disease-specific and general RSQMs. Pneumonia was chosen as a representative condition because it is commonly encountered with established clinical practice guidelines. However, given that the most common conditions in internal medicine have relatively low prevalence (approximately 5%),^9^ we also developed general RSQMs applicable to all admissions.

Using the CVF as a conceptual framework, 1 author (B.T.) drafted an initial list of RSQMs derived from clinical practice guidelines and existing quality measures in internal medicine. Proposed RSQMs were presented to the research team for iterative refinement until a consensus list had been generated according to numerous criteria (eAppendix 1 in Supplement 1). Seven RSQMs and 2 positive controls ultimately proposed for this study are defined in eTable 1 in Supplement 1, with the corresponding clinical guidelines described in eTable 2 in Supplement 1.

### Clinical and Residential Data

Clinical and resident data were extracted from the GEMINI Medical Education (MedED) database, for which the methodology and design have been previously described.^16,17^ This database combines existing clinical data from GEMINI and 10 years of call schedules (2010-2019) showing which senior resident covered overnight call for the inpatient internal medicine service. Although all included residents were senior (postgraduate year ≥2), call schedules did not specify training level or other identifying characteristics (ie, age, sex, race and ethnicity). A study time frame of July 1, 2010, to December 31, 2019, was selected based on data availability from both GEMINI and resident call schedules.

### Statistical Analysis

The data analysis was performed between March 1, 2024, and February 23, 2026. Descriptive statistics were calculated at the resident level for each RSQM. For each resident, we calculated an order proportion for each measure, with the numerator being the number of times the specific care was ordered (antibiotics, imaging) or performed (blood work, transfusions) and the denominator being the total number of eligible patients. Residents were then rank ordered by increasing order proportion for each RSQM and stratified into quartiles. We report the median and interquartile range of individual resident order proportions. To provide context for scale, patient-level order proportions are also reported in data tables. Although primary analyses were conducted at a resident level (resident order proportions), post hoc analyses were performed at a patient level. Race and ethnicity were not collected at the individual level in this study. However, to contextualize the diversity of the patient population, we reported area-level sociodemographic proxies (eg, neighborhood visible minority population composition and income quintile) derived from Canadian administrative data based on patients’ residential postal codes. Consistent with prior studies of physician care variation, the maximum standardized mean difference was used to determine imbalance between groups as signified by values greater than 0.1.^18^ All statistical analyses were performed using R, version 4.4.1 (R Foundation for Statistical Computing).

Based on our clinical workflows, we defined specific attribution time windows (eTable 3 in Supplement 1) for different CVF categories. Discretionary care and low-value care RSQMs included only postadmission orders (ie, orders at or after the time of admission until 8 am at the end of overnight call), which we expected to better represent the care of senior internal medicine residents rather than ED physicians. The evidence-based care measure ordering first-line antibiotics for pneumonia included orders from ED presentation until 8 am at the end of overnight call (admission day 0). Positive control measures also encompassed orders on admission day 0, given that these processes are typically initiated by ED physicians.

To evaluate the influence of contextual factors on resident practice, we also analyzed RSQMs according to the hospital where each call shift occurred and across resident cohorts during the study period. For the temporal analysis, RSQMs were compared between residents divided into 3 nonoverlapping groups based on the date of their first shift in GEMINI MedED, a surrogate for the start of their senior resident training. We used the Kruskal-Wallis test to evaluate differences in RSQMs between hospitals and over time. For one post hoc analysis regarding resident encounter volumes, we used the Brown-Forsythe test to evaluate for a significant change in variance between the 2 groups. The threshold for statistical significance was set at *P* < .05.

## Results

### Study Population

**Figure 1.**
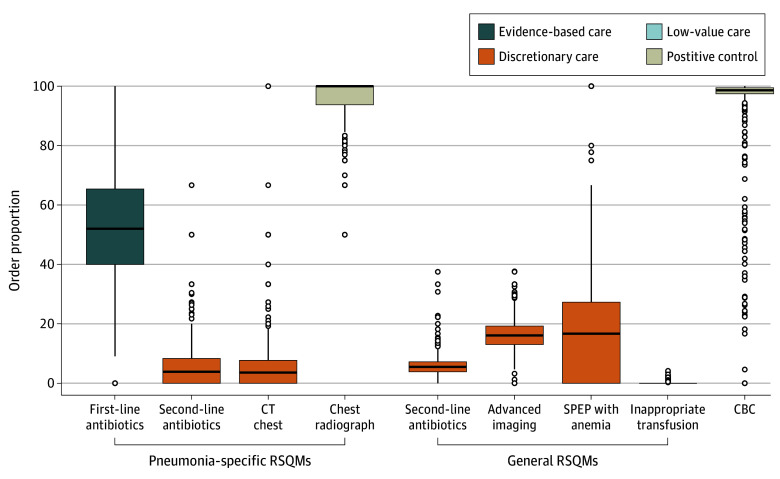
Box-and-Whisker Plots of Resident-Sensitive Quality Measure (RSQM) Median Ordering Proportions and Variation Grouped by the Care Variation Framework The upper edge of each box represents the 75th percentile, while the lower edge represents the 25th percentile. The height of each box represents the IQR. The horizontal line in each box represents the median (50th percentile). The bounds of the upper and lower whiskers represent 1.5 times the IQR. Individual circles denote outliers defined as values below quartile 1 minus 1.5 times the IQR or above quartile 3 plus 1.5 times the IQR. CBC indicates complete blood count; CT, computed tomography; SPEP, serum protein electrophoresis.

This study linked 793 senior internal medicine residents to 132 291 patient admissions (median [IQR] age, 70 [55-83] years; 49.4% female and 50.6% male) over a 10-year period (2010-2019).^16,17^ Resident data are summarized in eTable 4 in Supplement 1 and patient data in Table 1. Senior residents on call for general internal medicine performed a median (IQR) of 187 (89-228) patient admissions, including a median (IQR) of 18 (10-24) admissions for pneumonia. We report results as the median and IQR resident order proportions, with indicators categorized according to the CVF categories and summarized in Table 2 and Figure 1, with maximum standardized mean differences reported in eAppendix 2 in Supplement 1.

**Table 1.** Characteristics of Patients Treated by Senior Internal Medicine Residents During Overnight Call

Characteristic	Patients, No. (%)	
	All admissions (N = 132 291)	Admissions with pneumonia (n = 13 470)
Volume, No.		
Unique patients^a^	81 909	10 330
Total on-call shifts	16 205	NA
Demographics		
Age, median (IQR), y	70 (55-83)	75 (63-85)
Sex		
Female	65 333 (49.4)	6113 (45.4)
Male	66 958 (50.6)	7357 (54.6)
Top 5 primary discharge diagnoses		
Pneumonia	6947 (5.3)	NA
Heart failure	6354 (4.8)	NA
Chronic obstructive pulmonary disease	5690 (4.3)	NA
Urinary tract infections	5340 (4.0)	NA
Neurocognitive disorders	3854 (2.9)	NA
Acuity		
Modified laboratory-based acute physiology score, median (IQR)^b^	14 (6-25)	16 (6-27)
In-hospital death		
Total	7344 (5.6)	653 (4.9)
Within 48 h	1091 (0.8)	127 (0.9)
Medical complexity		
Overall Charlson Comorbidity Index score^c^		
0	67 929 (51.4)	5081 (37.7)
1	21 061 (15.9)	4089 (30.4)
≥2	43 301 (32.7)	4300 (31.9)
Previous 30-d hospitalization	11 858 (9.1)	1138 (8.5)
Social determinants of health		
Disability per *ICD-10* code	24 605 (18.6)	2081 (15.5)
From highest quintile visible minority neighborhoods	25 556 (20.0)	2252 (17.4)
From lowest income quintile neighborhood	38 312 (29.9)	4122 (31.8)

Abbreviations: *ICD-10*, *International Statistical Classification of Diseases, Tenth Revision*; NA, not applicable.

^a^ Included only patients with a valid health card number; approximately 1% of patients lacked this information.

^b^ Maximum theoretical range of 0 to 256, with higher scores indicating greater acuity.

^c^ Scale of 0 to 37, with higher scores indicating greater medical complexity.

**Table 2.** Order Proportions and Variation in RSQMs

Measure	Resident order proportion, median (IQR), %^a^	Maximum SMD^b^	Patient-level order proportion, No./total No. (%)	RSQM classification
**Pneumonia-specific measures**				
% First-line antibiotics order	52 (40-65.4)	1.0	7081/13 470 (52.6)	Evidenced-based care
% Second-line antibiotics order	3.8 (0-8.3)	0.7	1444/13 470 (10.7)	Discretionary care
% Chest computed tomography order	3.6 (0-7.7)	0.5	1909/13 470 (14.2)	Discretionary care
% Chest radiography order	100 (93.8-100)	NA	12 907/13 470 (95.8)	Positive control
**General measures**				
% Second-line antibiotics order	5.5 (3.8-7.2)	0.3	7514/132 291 (5.7)	Discretionary care
% Advanced imaging order^c^	16.1 (13-19.2)	0.3	21 220/132 291 (16.0)	Discretionary care
% SPEP collected with anemia	16.7 (0-27.3)	0.2	1261/100 384 (1.3)	Discretionary care
% Potentially inappropriate transfusions	0 (0-0)	NA	242/112 269 (0.2)	Low-value care
% CBC collected	98.6 (97.5-99.5)	0.6	126 865/132 291 (95.9)	Positive control

Abbreviations: CBC, complete blood count; NA, not applicable; RSQM, resident-sensitive quality measure; SMD, standardized mean difference; SPEP, serum protein electrophoresis.

^a^ Resident-level median order proportions and overall patient-level event counts are reported to provide context for scale and variation.

^b^ The maximum SMD for each measure was calculated using the 2 quartiles that had the largest pairwise SMD for each variable (calculated as the mean difference divided by pooled standard deviation). Standardized mean differences greater than 0.1 are considered meaningful markers of imbalance.

^c^ Including computed tomography, magnetic resonance imaging, and ultrasonography.

### Positive Controls, Low-Value Care, and Discretionary Care

As anticipated for the 2 positive controls, the median resident order proportion was 100% for chest radiographs for pneumonia (IQR, 93.8%-100%) and 98.6% for complete blood count collections across all admissions (IQR, 97.5%-99.5%). For low-value care (1 RSQM) across all admissions, residents ordered potentially inappropriate red blood cell transfusions (with pretransfusion hemoglobin levels >80 g/L) for a median of 0% (IQR, 0%-0%) of admissions (range, 0%-4.2% of admissions).

For discretionary care (5 RSQMs), RSQMs showed mixed findings among residents. Two general care RSQMs occurred with relatively high frequency and mixed variability: advanced imaging ordering (median, 16.1%; IQR, 13%-19.2%) and serum protein electrophoresis collections (median, 16.7%; IQR, 0%-27.3%). However, second-line antibiotic ordering across all admissions was the least variable of all measures in this category (median, 6%; IQR, 4%-7%). In contrast, disease-specific RSQMs for pneumonia occurred with relatively low frequency and variability, including second-line antibiotic ordering (median, 3.8%; IQR, 0%-8.3%) and chest computed tomography ordering (median, 3.6%; IQR, 0%-7.7%).

### Evidence-Based Care

**Figure 2.**
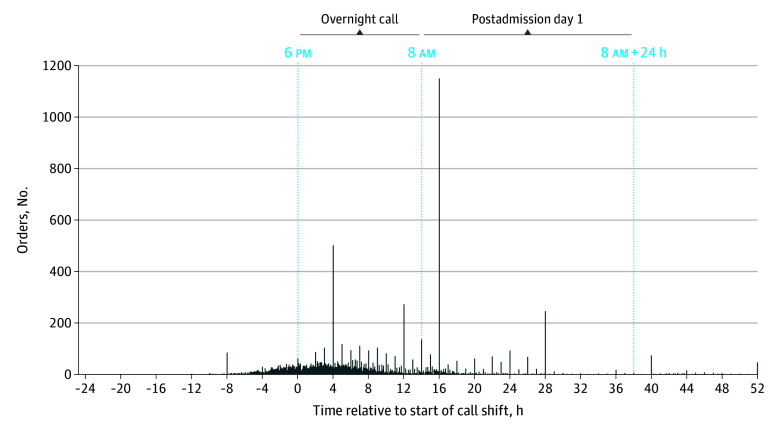
Histogram of First-Line Antibiotic Orders in Pneumonia Relative to the Start of the Overnight Call Shift The blue dashed lines represent the start and end of the overnight call shift. Negative numbers represent orders entered before the call shift. Positive numbers represent orders entered after the start of the call shift.

For the evidence-based care RSQM, residents ordered first-line antibiotics for pneumonia for a median of 20% of admissions (IQR, 11%-32% of admissions) when restricted to postadmission orders. Because this proportion was lower than expected,^19^ we conducted 4 post hoc analyses at a patient level to explore contributing factors (preadmission orders, orders after the call shift, patient length of stay, and resident encounter volumes). First, we hypothesized that the ED physician ordered first-line antibiotics prior to referral (and, therefore, hospital admission) in a subset of cases. To explore this hypothesis, we extended the attribution time window to the time of initial ED presentation. Of 13 470 admissions, the proportion of pneumonia admissions with first-line antibiotics ordered was then 52.6% (n = 7081), with 33.8% (n = 4549) of orders entered before admission, potentially by ED physicians (eTable 5 in Supplement 1). We further explored whether first-line antibiotics were also ordered after the senior resident’s call shift. For an additional 23.2% (n = 3124) of admissions, the initial order for first-line antibiotics occurred during the 24-hour period after the call shift, from 8 am to 8 am (postadmission day 1), bringing the cumulative proportion to 75.8% (n = 10 205) from ED presentation through postadmission day 1 and 81.6% (n = 10 990) until the time of discharge (Table 3). A more detailed time analysis showed that most orders on postadmission day 1 were clustered within 2 hours after the call shift, between 8 and 10 am (Figure 2). Overall, the patient-level order proportion was highly sensitive to the attribution time window, ranging from 22.5% (n = 3027) to 75.8% (n = 10 205) depending on the cutoffs applied. As previously described, we ultimately reported resident-level first-line antibiotic orders for pneumonia from ED presentation until the end of the call shift at 8 am (Table 2; Figure 1), recognizing that orders entered by ED physicians could appropriately obviate the need for duplicate resident orders. Overall, residents ordered these antibiotics for a median of 52.0% of admissions (IQR, 40.0%-65.4% of admissions).

**Table 3.** Antibiotic Ordering for Pneumonia on Admission Day 0 and Subsequent Admission Periods (n = 13 470)

Measure	Admission day 0, % (No.)^a^	Postadmission day, % (No.)					
		1^b^	2	3	4	5	6 to Discharge
**First-line antibiotics**							
% Ordered^c^	52.6 (7081)	23.2 (3124)	2.5 (337)	1.3 (173)	0.7 (93)	0.4 (60)	0.9 (122)
% Cumulative^d^	52.6 (7081)^e^	75.8 (10 205)^e^	78.3 (10 542)	79.6 (10 715)	80.3 (10 808)	80.7 (10 868)	81.6 (10 990)^e^
**Second-line antibiotics**							
% Ordered^c^	10.7 (1443)	3.8 (510)	1.4 (190)	0.7 (98)	0.5 (63)	0.2 (33)	1.4 (182)
% Cumulative^d^	10.7 (1443)^e^	14.5 (1953)^e^	15.9 (2143)	16.6 (2241)	17.1 (2304)	17.3 (2337)	18.7 (2519)^e^
**Either first-line or second-line antibiotics**							
% Ordered^c^	58.7 (7910)	22.5 (3026)	2.1 (277)	0.8 (103)	0.4 (50)	0.3 (35)	0.6 (78)
% Cumulative^d^	58.7 (7910)^e^	81.2 (10 936)^e^	83.2 (11 213)	84.0 (11 316)	84.4 (11 366)	84.6 (11 401)	85.2 (11 479)^e^

^a^ Admission day 0 is defined as the period from emergency department presentation until 8 am at the end of overnight call.

^b^ Postadmission day 1 is defined as the 24-hour period starting at 8 am after overnight call, with postadmission days 2, 3, etc each representing subsequent 24-hour time intervals.

^c^ Refers to the proportion of orders entered for the first time within each period.

^d^ Refers to the cumulative proportion of orders entered by the end of each period.

^e^ A key period subset.

We also considered that longer hospital stays could be associated with admission presentations differing from the discharge diagnosis (eg, a patient discharged with pneumonia did not initially present with pneumonia) and whether residents with lower admission volumes had order proportions disproportionately skewed toward extremes. However, analyses based on patient length of stay and resident pneumonia encounter volumes minimally influenced the results, as shown in eTables 6 and 7 in Supplement 1.

### Training Site and Time Trends

All measures except potentially inappropriate transfusions showed statistically significant differences in resident order proportion based on training site (hospitals) (eTable 8 in Supplement 1). For example, the proportion of serum protein electrophoresis collections varied by hospital, ranging from a median of 0% (IQR, 0%-16.7%) to 25.0% (IQR, 0%-50.0%) (*P* < .001). Several measures revealed statistically significant differences in use based on resident cohorts over time, such as second-line antibiotic ordering, which increased from a median of 3.0% (IQR, 0%-7.7%) among residents whose first shift occurred in 2010 to 2013 to 4.3% (IQR, 0%-9.1%) among those whose first shift was in 2016 to 2019 (*P* = .01) (eTable 9 in Supplement 1).

## Discussion

This retrospective cohort study examined the feasibility of developing resident-level quality measures from EHR data to inform GME. We outline a 5-step approach that could be applied in other training programs to develop and evaluate RSQMs. Although RSQMs show potential, our findings highlight challenges of attribution and statistical reliability that should be considered before RSQMs are applied in GME. To support the development of RSQMs from EHR data, our approach followed 5 key steps: (1) identify common resident-managed conditions, review clinical guidelines, and draft candidate RSQMs (disease-specific and general care); (2) define how to attribute patient care to residents (eg, time-based windows, ordering provider), acknowledging the limitations of the selected approach; (3) characterize practice variation using EHR data, comparing observed variation against expected patterns using the CVF; (4) investigate unexpected variation patterns; and (5) decide whether and how RSQMs should be used at the individual resident level and/or the aggregated program level.

When using the CVF (step 3) in this study, for 6 of 7 RSQMs, the observed variation aligned with expected patterns. However, for 1 RSQM, the evidence-based measure of first-line antibiotic ordering for pneumonia, antibiotic ordering rates varied widely depending on how the attribution time window was defined. We initially restricted first-line antibiotic orders for pneumonia to those placed after admission, reasoning that these were the most likely to represent resident-initiated treatment. This approach yielded a median resident order proportion of 20%, far lower than expected from local experience.^19^ Expanding the attribution window to include preadmission orders, potentially entered by the ED physician, increased this proportion to 52%. This finding highlights the methodological challenge of defining attribution rules for inpatient RSQMs (step 2), in which patients encounter multiple physicians across care transitions and care decisions can be interdependent. That we could not reconcile unexpected variation patterns through post hoc analyses (step 4) led us to reconsider using this RSQM at the individual resident level. Alternative attribution approaches, such as using the ordering physician in EHR data, have similar limitations and risks of misattribution. For example, overnight orders may be entered by junior residents at the direction of the senior resident, meaning that these orders would not be attributed to the supervising senior resident. Ultimately, the ideal approach to attributing patient care to individual residents is an unresolved challenge, with no criterion standard identified as in a recent review.^12^

Even if we were able to overcome attribution challenges, our study also raised statistical reliability concerns for individual-level RSQMs. Individual residents had relatively small numbers of disease-specific encounters (eg, median [IQR] pneumonia admissions, 18 [10-24]), in keeping with prior studies of attending internal medicine physician case mix.^9^ These low sample sizes coupled with infrequent event rates of certain RSQMs (eg, <5% for chest computed tomography and second-line antibiotic ordering for pneumonia) raised reliability concerns that observed variation could represent measurement error as opposed to true interresident differences. Although beyond the scope of this descriptive study, future work could aim to explore the underlying sources of observed variation through statistical approaches such as variance partitioning (step 4). Such approaches may ultimately identify that it may be more statistically robust to rely on general RSQMs, encompassing all patient admissions for which patient volumes were nearly 10 times higher than pneumonia in this study.

Challenges with attribution and statistical reliability raise concerns about the feasibility of using individual-level RSQMs for inpatient internal medicine care. Prior studies of RSQMs in internal medicine have focused on outpatient settings, in which attribution of care to residents is more direct.^20^ Alternatively, examining RSQM performance at a program level has been shown to increase measurement reliability while still offering educationally relevant insights (step 5).^21^ For example, this study identified a large cluster of first-line antibiotic orders for pneumonia entered between 8 and 10 am after call, which may reflect the input of attending physician case review. Hence, although these orders are not attributable to the overnight resident, this finding may still offer program-level insight into on-call resident decision-making. Other program-level use cases could be to examine differences in resident practice patterns between hospital sites or cohorts over time (eg, second-line antibiotic orders as a signal for antibiotic stewardship). Such data could support numerous program-level educational efforts, such as curriculum development or continuous quality improvement, in line with accreditation standards.

An ideal future use case for RSQMs in GME would be to benchmark performance over time by monitoring individual resident and/or cohort progress. In this study, given the lack of demographic data on residents’ postgraduate year and the limited number of patient encounters per resident, it was not feasible to assess individual resident changes in performance over time. Considering these limitations, we believe that the specific RSQMs we evaluated are currently best suited for program-level applications, while limiting individual-level use to exploratory, formative purposes. The 5-step approach we outlined offers a guide for the continued development and evaluation of RSQMs in other contexts.

### Limitations

This study had some limitations. We focused on senior internal medicine residents during overnight internal medicine call at a single Canadian institution, limiting generalizability to other training environments. As an observational study, the relative contributions of resident factors and contextual influences, such as patient variation and hospital workflows, to the observed variation remains undefined. Finally, our findings should be interpreted in the context of the limitations of time-based attribution and statistical reliability of individual-level RSQMs.

## Conclusions

This cohort study of senior internal medicine residents in a Canadian residency program outlined an approach to developing and evaluating RSQMs using readily available EHR data. Although RSQMs have the potential to support GME, their use in inpatient internal medicine contexts may be more appropriate at the program level due to unresolved issues associated with attribution and statistical reliability.

## Notes

Supplement 1. **eAppendix 1.** Criteria for Selecting Resident-Sensitive Quality Measures **eTable 1.** Definitions of Resident-Sensitive Quality Measures (RSQMs) for Pneumonia-Specific and General Clinical Care **eTable 2.** Clinical Guidelines Used to Define Each RSQM **eTable 3.** Definitions of Key Periods in a Patient’s Hospitalization in GEMINI MedED **eTable 4.** Summary of Resident Data in GEMINI MedED **eAppendix 2.** Calculation and Reporting of Maximum Standardized Mean Differences **eTable 5.** Post Hoc Analysis of RSQM Performance Stratified Before Admission, After Admission, and Admission Day 0 **eTable 6.** Post Hoc Analysis of Pneumonia RSQM Performance, Comparing All Admissions vs Admissions Less Than 14 Days Length of Stay **eTable 7.** Post Hoc Analysis of Pneumonia RSQM Ordering Proportions, Comparing All Residents and Residents in the Highest Quartile of Pneumonia Admission Volumes **eTable 8.** Descriptive Statistics for Each RSQM Stratified by Hospital **eTable 9.** Descriptive Statistics for Each RSQM Stratified by Periods
eReferences.

Supplement 2.
Data Sharing Statement
