# Supplementary material for: Developing Resident-Sensitive Quality Measures for Internal Medicine
Source: JAMA Netw Open. 2026 May 8;9(5):e2611700. doi: 10.1001/jamanetworkopen.2026.11700 (PMC13156782; doi:10.1001/jamanetworkopen.2026.11700)
Supplement: Supplement 1. — eAppendix 1. Criteria for Selecting Resident-Sensitive Quality Measures eTable 1. Definitions of Resident-Sensitive Quality Measures (RSQMs) for Pneumonia-Specific and General Clinical Care eTable 2. Clinical Guidelines Used to Define Each RSQM eTable 3. Definitions of Key Periods in a Patient’s Hospitalization in GEMINI MedED eTable 4. Summary of Resident Data in GEMINI MedED eAppendix 2. Calculation and Reporting of Maximum Standardized Mean Differences eTable 5. Post Hoc Analysis of RSQM Performance Stratified Before Admission, After Admission, and Admission Day 0 eTable 6. Post Hoc Analysis of Pneumonia RSQM Performance, Comparing All Admissions vs Admissions Less Than 14 Days Length of Stay eTable 7. Post Hoc Analysis of Pneumonia RSQM Ordering Proportions, Comparing All Residents and Residents in the Highest Quartile of Pneumonia Admission Volumes eTable 8. Descriptive Statistics for Each RSQM Stratified by Hospital eTable 9. Descriptive Statistics for Each RSQM Stratified by Periods eReferences. [file jamanetwopen-e2611700-s001.pdf]

Supplemental Online Content

Tang B, Lam ACL, Wankiewicz M, et al. Developing resident-sensitive quality measures for internal medicine. *JAMA Netw Open*. 2026;9(5):e2611700.
doi:10.1001/jamanetworkopen.2026.11700

eAppendix 1. Criteria for Selecting Resident-Sensitive Quality Measures

eTable 1. Definitions of Resident-Sensitive Quality Measures (RSQMs) for Pneumonia-Specific and General Clinical Care

eTable 2. Clinical Guidelines Used to Define Each RSQM

eTable 3. Definitions of Key Periods in a Patient's Hospitalization in GEMINI MedED

eTable 4. Summary of Resident Data in GEMINI MedED

eAppendix 2. Calculation and Reporting of Maximum Standardized Mean Differences

eTable 5. Post Hoc Analysis of RSQM Performance Stratified Before Admission, After Admission, and Admission Day 0

eTable 6. Post Hoc Analysis of Pneumonia RSQM Performance, Comparing All Admissions vs Admissions Less Than 14 Days Length of Stay

eTable 7. Post Hoc Analysis of Pneumonia RSQM Ordering Proportions, Comparing All Residents and Residents in the Highest Quartile of Pneumonia Admission Volumes

eTable 8. Descriptive Statistics for Each RSQM Stratified by Hospital

eTable 9. Descriptive Statistics for Each RSQM Stratified by Periods

eReferences

This supplemental material has been provided by the authors to give readers additional information about their work.

© 2026 Tang B et al. *JAMA Network Open*.

eAppendix 1. Criteria for Selecting Resident-Sensitive Quality Measures

1. Established standards for selecting quality measures: Relevance to patient care, scientific validity, and feasibility of measurement.[1] In particular, feasibility considered the measurement limitations of GEMINI, which does not include select clinical orders such as code status and vital signs.
2. Alignment with previously proposed quality measures in IM[2-7] and physician practice feedback reports[8,9]
3. Alignment with clinical practice guidelines.[10-12] Given the ten year timeframe of this study, we aimed to select RSQMs with consistent guideline recommendations throughout the study period.
4. Potential to identify practice variation: Measures were proposed to represent the different categories of the CVF
5. Resident attributability
6. Data availability in GEMINI MedED

© 2026 Tang B et al. *JAMA Network Open*.

eTable 1. Definitions of Resident-Sensitive Quality Measures (RSQMs) for Pneumonia-Specific and General Clinical Care

Measure	Numerator	Denominator	Attribution time window*	RSQM Classification
**Pneumonia-Specific Measures**				
**% First-line antibiotics ordered**	1. Ordered beta-lactam (ceftriaxone, cefotaxime, ceftaroline, or amoxicillin-clavulanic acid)<br>OR<br>2. Ordered respiratory fluoroquinolone (levofloxacin or moxifloxacin)	All pneumonia admissions	Admission day 0	Evidenced-Based Care
**% Second-line antibiotics ordered**	Ordered vancomycin, linezolid, pipercillin-tazobactam, or carbapenem (ertapenem, imipenem, meropenem)	All pneumonia admissions	After admission	Discretionary Care
**% CT chest ordered**	Ordered CT chest (high-resolution CT chest, CT pulmonary angiogram, or CT angiogram chest)	All pneumonia admissions	After admission	Discretionary Care
**% CXR ordered**	Ordered chest x-ray	All pneumonia admissions	Admission day 0	Positive Control
**General Measures**				
**% Second-line antibiotics ordered**	Ordered vancomycin, linezolid, pipercillin-tazobactam, or carbapenem (ertapenem, imipenem, meropenem)	All admissions	After admission	Discretionary Care
**% Advanced imaging ordered (CT, MRI, US)**	Ordered advanced imaging (CT, MRI, US)	All admissions	After admission	Discretionary Care
**% SPEP collected with anemia**	SPEP sample collected from patient	All admissions with anemia**	After admission	Discretionary Care
**% Potentially inappropriate transfusions**	Administration of red blood cell transfusion	Admissions with all pre-transfusion hemoglobin levels >80 g/L (within 48 hours of admission)	After admission	Low-Value Care
**% CBC collected**	CBC sample collected from patient	All admissions	Admission day 0	Positive Control

*After admission refers to the time period from an admission order until 8AM at the end of overnight call; admission day 0 refers to the period from ED presentation until 8AM at the end of overnight call
**Anemia was defined as a hemoglobin <130 g/L in men and <120 g/L in women
CBC: Complete blood count; CT: Computed tomography; CXR: Chest x-ray; MRI: Magnetic resonance imaging; SPEP: Serum protein electrophoresis; US: Ultrasound

© 2026 Tang B et al. *JAMA Network Open*.

eTable 2. Clinical Guidelines Used to Define Each RSQM

Measure	Guideline Recommendation	Guideline
**Pneumonia-Specific RSQMs (n=5)**		
**% First-line antibiotics ordered**	Standard regimen for community-acquired pneumonia (CAP) includes either beta-lactam or respiratory fluroquinolone. Combination therapy with macrolide recommended when beta-lactams used.	ATS/IDSA 2019 [13]
	Monotherapy with beta-lactams or respiratory fluroquinolone recommended. Use of macrolide not routinely recommended.	Ontario Toronto Central LHIN 2016 [14]
	No significant difference in mortality or treatment failure in hospitalized patients receiving monotherapy (e.g., beta-lactam or quinolone) versus combination therapy (e.g., beta-lactam plus macrolide) for CAP.	Health Quality Ontario 2013 [15]
**% Second-line antibiotics ordered**	Add coverage for Methicillin-resistant Staphylococcus aureus (MRSA) and/or Pseudomonas aeruginosa based on prior respiratory isolates.	ATS/IDSA 2019 [13]
	Add vancomycin if MRSA colonization known/suspected in critically ill patients.	Health Quality Ontario 2013 [15]
**% CT chest ordered**	CT scan is not recommended for <u>diagnosis</u> of CAP.	Health Quality Ontario 2014 [16]
	CT scan can be valuable in the <u>management</u> of nonresponding CAP.	ATS/IDSA 2007 [17]
**% CXR ordered**	Recommended for all patients with symptoms of CAP to confirm the diagnosis.	Health Quality Ontario 2014 [16]
**General RSQMs (n=5)**		
**% Second-line antibiotics ordered**	Disease-specific treatment guidelines, pathways, and algorithms should guide local treatment recommendations.	Government of Canada 2017 [18]; Public Health Ontario 2016 [19]
	Broad spectrum antibiotics may be defined as “a combination of antibiotics which act against a wide range of disease-causing bacteria”; these included cephalosporins, aminoglycosides, vancomycin, and carbapenems.	Cochrane Review 2010 [20]
**% Advanced imaging ordered (CT, MRI, US)**	Context-specific recommendations from Choosing Wisely Initiative regarding overuse of diagnostic imaging (e.g., avoid imaging for lower-back pain unless red flags are present).	Canadian Institute for Health Information 2022 [21]; Choosing Wisely 2012 [22]
**% SPEP collected with anemia**	Not recommended in asymptomatic patients in the absence of otherwise unexplained hypercalcemia, renal insufficiency, anemia, or lytic bone lesions.	Choosing Wisely Canada 2023

© 2026 Tang B et al. *JAMA Network Open*.

	Routine monoclonal gammopathy screening in the evaluation of anemia has low clinical utility.	Maberry et al. 2023 [23]
	May be inappropriate to repeat SPEP when already performed within the past 3 months; 6.3% of SPEP tests in Ontario were potentially inappropriate.	Chamia et al. 2017 [24]
**% Potentially inappropriate transfusions**	"Don't transfuse red blood cells for arbitrary hemoglobin or hematocrit thresholds in the absence of symptoms, active coronary disease, heart failure or stroke."	Canadian Institute for Health Information 2022 [21]; Choosing Wisely Canada 2022 [25]
**% CBC collected**	N/A	N/A

CBC: Complete blood count; CT: Computed tomography; CXR: Chest x-ray; MRI: Magnetic resonance imaging; US: Ultrasound; SPEP: Serum protein electrophoresis

© 2026 Tang B et al. *JAMA Network Open*.

eTable 3. Definitions of Key Periods in a Patient's Hospitalization in GEMINI MedED

Definition	Time period	Duration (hours)
Overnight call "attribution time window"	6PM until 8AM the following day	14
Before admission	ED presentation until admission order	Variable
After admission	Admission order until 8AM at the end of overnight call	Variable
Admission day 0	ED presentation until 8AM at the end of overnight call	Variable
Post-admission day 1	8AM at the end of overnight call until 8AM the following day	24

© 2026 Tang B et al. *JAMA Network Open*.

eTable 1. Summary of Resident Data in GEMINI MedED

Variable	Sample Size (IQR)
Residents	793
Hospital sites	5
Academic years	10
Median Shifts per Resident (IQR)	23 (11-28)
Median Admissions per Shift (IQR)	8 (6-10)
Median Admissions per Resident (IQR)	187 (89-228)
Median Pneumonia Admissions per Resident (IQR)	18 (10-24)

IQR: Interquartile range, reported as 25th percentile-75th percentile

© 2026 Tang B et al. *JAMA Network Open*.

eAppendix 2. Calculation and Reporting of Maximum Standardized Mean Differences

The presence of imbalance in our resident cohort's clinical practice was determined by calculating maximum standardized mean difference, commonly referred to as "maximum standardized difference" (MSD). This approach was derived from prior observational studies which used MSD to compare mean differences between groups and studies of attending physician care variation from our research team [9,26]. For each RSQM, we calculated the standardized mean difference between each quartile (e.g., between quartiles 1 and 2, 1 and 3, 1 and 4, 2 and 3, 2 and 4, etc.). Standardized mean difference was calculated as the mean difference between quartiles divided by the pooled standard deviation across both quartiles. The largest standardized mean difference across all pairwise comparisons for each RSQM was referred to as the MSD. Values higher than 0.1 were considered to denote imbalance between groups [21].

Pneumonia-specific RSQMs

The MSD was >0.1 for ordering of first and second-line antibiotics as well as CT chest, indicating an imbalance in ordering proportion between residents. The MSD could not be calculated for chest x-ray ordering as multiple quartiles had identical ordering proportions – calculating the pairwise standardized difference for these identical quartiles was not possible, precluding calculation of MSD.

General RSQMs

The MSD was >0.1 for four of five general RSQMs (second-line antibiotics ordering, advanced imaging ordering, SPEP collections with anemia, and CBC collections), indicating an overall imbalance in ordering proportions between residents. The MSD could not be calculated for administrations of potentially inappropriate transfusions as multiple quartiles had identical ordering proportions.

© 2026 Tang B et al. *JAMA Network Open*.

eTable 2. Post Hoc Analysis of RSQM Performance Stratified Before Admission, After Admission, and Admission Day 0

Measure	Ordered before admission, No. (%)	Ordered after admission, No. (%)	Ordered on admission day 0 (before or after admission), No. (%)	Total admissions	RSQM classification
**Pneumonia-Specific RSQMs**					
**% First-line antibiotics ordered**	4549 (33.8)	3027 (22.5)	7081 (52.6)	13,470	Evidenced-Based Care
**% CXR ordered**	12,744 (94.6)	655 (4.9)	12,907 (95.8)	13,470	Positive Control
**General RSQMs**					
**% CBC collected**	124,678 (94.2)	54,257 (41.0)	126,865 (95.9)	132,291	Positive Control

© 2026 Tang B et al. *JAMA Network Open*.

eTable 3. Post Hoc Analysis of Pneumonia RSQM Performance, Comparing All Admissions vs Admissions Less Than 14 Days Length of Stay

13,470 admissions with pneumonia were included in the analysis with all LOS, while 12,387 admissions had a LOS <14 days

Measure	Median performance (all LOS), %	Median performance (LOS <14 days), %	RSQM classification
**% First-line antibiotics ordered**	52.0	52.6	Evidenced-Based Care
**% Second-line antibiotics ordered**	3.8	3.3	Discretionary Care
**% CT chest ordered**	3.6	2.9	Discretionary Care
**% CXR ordered**	100	100	Positive Control

LOS: Length of stay

© 2026 Tang B et al. *JAMA Network Open*.

eTable 4. Post Hoc Analysis of Pneumonia RSQM Ordering Proportions, Comparing All Residents and Residents in the Highest Quartile of Pneumonia Admission Volumes

Measure	Residents in 1st to 3rd quartile of pneumonia admissions		Residents in 4th quartile of pneumonia admissions		p value*	RSQM classification
	Median, %	IQR, %	Median, %	IQR, %		
**% First-line antibiotics ordered**	51.1 (4077/7870)	35.9-66.7	52.0 (3000/5600)	46.4-62.1	0.80	Evidenced-Based Care
**% Second-line antibiotics ordered**	0 (433/7870)	0-8.3	4.0 (322/5600)	3.1-7.7	0.06	Discretionary Care
**% CT chest ordered**	0 (395/7870)	0-8.2	3.8 (297/5600)	2.9-7.7	0.13	Discretionary Care
**% CXR ordered**	100 (7560/7870)	94.1-100	96.4 (5347/5600)	92.9-100	0.38	Positive Control

*The Brown-Forsythe test was used to determine a significant change in variance between the two groups; p values of < .05 would meet the threshold for statistical significance.

© 2026 Tang B et al. *JAMA Network Open*.

eTable 5. Descriptive Statistics for Each RSQM Stratified by Hospital (A-E)

	A		B		C		D		E		MSD[A]
**Volumes**											
**Total pneumonia admissions**	2385		2107		2549		3193		3236		0.1
**Total patient admissions**	24657		23271		24657		32720		26986		0.3
**Pneumonia-Specific RSQMs (n=4)**											
	**Median**	**IQR**	**Median**	**IQR**	**Median**	**IQR**	**Median**	**IQR**	**Median**	**IQR**	**p value[B]**
**% First-line antibiotics order**	33.3	0-50	40	25-50	63.6	38.5-84.6	66.7	50-80	58.1	36.6-80	< .01 *
**% Second-line antibiotics order**	0	0-7.3	0	0-0	0	0-9.1	0	0-12.5	0	0-0	< .01 *
**% CT chest order**	0	0-0	0	0-0	0	0-5.9	0	0-12.5	0	0-8.3	< .01 *
**% CXR order**	100	100-100	100	95.5-100	100	100-100	100	87.5-100	100	100-100	< .01 *
**General RSQMs (n=5)**											
**% Second-line antibiotics order**	5.9	2.5-8.9	2.1	0-4.3	5.5	0-9.1	7.7	4.6-10.4	3.1	0-5.4	< .01 *
**% Advanced imaging order (CT, MRI, US)**	14.9	11.1-19.4	12.0	7.9-15.4	18.2	13.3-22.9	17.8	13.7-22.4	15.7	11.1-20.4	< .01 *
**% SPEP collected with anemia**	25	0-50	8.3	0-32.1	10	0-28.6	0	0-20	0	0-16.7	< .01 *
**% Potentially inappropriate transfusions**	0	0-0	0	0-0	0	0-0	0	0-0	0	0-0	0.06
**% CBC collected**	100	98.8-100	100	98.9-100	100	98.2-100	97.5	94.7-100	100	97.9-100	< .01 *

*Statistically significant p value

[A]MSD (Maximum Standardized Difference): The standardized mean difference (SMD) between the hospitals with the highest and lowest proportion for each measure was reported, with bolded values indicating a meaningful level of imbalance.

[B]The Kruskal-Wallis test was used to calculate p-values, with a threshold of p < .05 for significance

IQR: Interquartile range

eTable 6. Descriptive Statistics for Each RSQM Stratified by Periods

The resident population was divided into three distinct groups based on the year of their first shift in GEMINI-MedED

	Jul 1, 2010 – Jun 30, 2013 (n=288)		Jul 1, 2013 – Jun 30, 2016 (n=227)		Jul 1, 2016 – Dec 31, 2019 (n=278)		MSD[A]
**Patient Volumes**							
**Total pneumonia admissions**	4204		4338		4928		0.4
**Total patient admissions**	41007		43824		47460		0.6
**Pneumonia-Specific RSQMs (n=4)**							
	**Median**	**IQR**	**Median**	**IQR**	**Median**	**IQR**	**p value[B]**
**% First-line antibiotics order**	53.3	33.3-66.7	52.3	43.9-62.1	51.9	41.7-65.8	0.7
**% Second-line antibiotics order**	3.0	0-7.7	4	0-7.7	4.3	0-9.1	0.1
**% CT chest order**	1.0	0-7.7	3.8	0-7.7	3.7	0-7.7	0.6
**% CXR order**	100	96-100	97.0	93.9-100	96.8	91.2-100	< .01 *
**General RSQMs (n=5)**							
**% Second-line antibiotics order**	5.1	3.2-6.9	5.7	4.4-7.1	5.8	3.9-7.4	< .01 *
**% Advanced imaging order (CT, MRI, US)**	16.6	13.8-20	15.5	12.5-18.1	16.0	13.0-19.3	0.01 *
**% SPEP collected with anemia**	16.7	0-30	15.4	0-25	16.7	6.3-27.3	0.45
**% Potentially inappropriate transfusions**	0	0-0.5	0	0-0	0	0-0	< .01 *[C]

© 2026 Tang B et al. *JAMA Network Open*.

% CBC collected	98.6	95.4-99.5	98.6	97.9-99.5	98.6	97.6-99.3	< .05 *

*Statistically significant p value
[A]MSD (Maximum Standardized Difference): The standardized mean difference (SMD) between the hospitals with the highest and lowest proportion for each measure was reported, with bolded values indicating a meaningful level of imbalance.
[B]The Kruskal-Wallis test was used to calculate p-values, with a threshold of $p < .05$ for significance
[C]Although the IQR for all three cohorts was similar, the overall distribution of data between cohorts still varied resulting in a statistically significant difference in variance between cohorts
IQR: Interquartile range

© 2026 Tang B et al. *JAMA Network Open*.

eReferences

1. Quentin W, Partanen VM, Brownwood I, Klazinga N. 3. Measuring healthcare quality. In: Busse R, Klazinga N, Panteli D, et al, eds. Improving Healthcare Quality in Europe: Characteristics, Effectiveness and Implementation of Different Strategies. European Observatory on Health Systems and Policies; 2019. Accessed January 30, 2024. https://www.ncbi.nlm.nih.gov/books/NBK549260/

2. Schumacher DJ, Martini A, Holmboe E, et al. Initial implementation of resident-sensitive quality measures in the pediatric emergency department: a wide range of performance. Acad Med. 2020;95(8):1248-1255. Medline:31913878 doi:10.1097/ACM.0000000000003147

3. Kinnear B, Kelleher M, Sall D, et al. Development of resident-sensitive quality measures for inpatient general internal medicine. J Gen Intern Med. 2021;36(5):1271-1278. Medline:33105001 doi:10.1007/s11606-020-06320-0

4. Schumacher DJ, Wu DTY, Meganathan K, et al. A feasibility study to attribute patients to primary interns on inpatient ward teams using electronic health record data. Acad Med. 2019;94(9):1376-1383. Medline:31460936 doi:10.1097/ACM.0000000000002748

5. Bonow RO, Ganiats TG, Beam CT, et al; American College of Cardiology Foundation; American Heart Association Task Force on Performance Measures; American Medical Association-Physician Consortium for Performance Improvement. ACCF/AHA/AMA-PCPI 2011 performance measures for adults with heart failure: a report of the American College of Cardiology Foundation/American Heart Association Task Force on Performance Measures and the American Medical Association–Physician Consortium for Performance Improvement. Circulation. 2012;125(19):2382-2401. Medline:22528524 doi:10.1161/CIR.0b013e3182507bec

6. Goldstein R, Kaplan A. Chronic obstructive pulmonary disease care in the community for adults. Health Quality Ontario. 2018. Accessed January 30, 2024. https://www.hqontario.ca/Portals/0/documents/evidence/quality-standards/qs-chronic-obstructive-pulmonary-disease-quality-standard-en.pdf

7. Rhew DC. Quality indicators for the management of pneumonia in vulnerable elders. Ann Intern Med. 2001;135(8 pt 2):736-743. Medline:11601957 doi:10.7326/0003-4819-135-8_Part_2-200110161-00013

8. Verma AA, Razak F. Lessons for hospital care from the first wave of COVID-19 in Ontario, Canada. Hosp Pract (1995). 2021;49(4):229-231. Medline:33832401 doi:10.1080/21548331.2021.1915657

© 2026 Tang B et al. *JAMA Network Open*.

9. Verma AA, Guo Y, Jung HY, et al. Physician-level variation in clinical outcomes and resource use in inpatient general internal medicine: an observational study. BMJ Qual Saf. 2021;30(2):123-132. Medline:32220936 doi:10.1136/bmjqs-2019-010425

10. Wedzicha JA, Miravitlles M, Hurst JR, et al. Management of COPD exacerbations: a European Respiratory Society/American Thoracic Society guideline. Eur Respir J. 2017;49(3):1600791. Medline:28298398 doi:10.1183/13993003.00791-2016

11. Mandell LA, Marrie TJ, Grossman RF, Chow AW, Hyland RH; The Canadian Community-Acquired Pneumonia Working Group. Canadian guidelines for the initial management of community-acquired pneumonia: an evidence-based update by the Canadian Infectious Diseases Society and the Canadian Thoracic Society. Clin Infect Dis. 2000;31(2):383-421. Medline:10987698 doi:10.1086/313959

12. Ezekowitz JA, O'Meara E, McDonald MA, et al. 2017 Comprehensive update of the Canadian Cardiovascular Society guidelines for the management of heart failure. Can J Cardiol. 2017;33(11):1342-1433. Medline:29111106 doi:10.1016/j.cjca.2017.08.022

13. Metlay JP, Waterer GW, Long AC, et al. Diagnosis and treatment of adults with community-acquired pneumonia. Am J Respir Crit Care Med. 2019;200(7):e45-e67. Medline:31573350 doi:10.1164/rccm.201908-1581ST

14. Management of Community-Acquired Pneumonia. Toronto Central Local Health Network. October 2016.

15. Monotherapy versus combination therapy for adults hospitalized for community-acquired pneumonia: a rapid review. Health Quality Ontario. November 2013. Accessed January 30, 2024. https://www.hqontario.ca/Portals/0/Documents/evidence/rapid-reviews/pneumonia-mono-vs-combo-therapy-131120-en.pdf

16. Health Quality Ontario; Ministry of Health and Long-Term. Quality-based procedures: clinical handbook for community-acquired pneumonia. Health Quality Ontario. February 2014. Accessed January 30, 2024.
https://www.hqontario.ca/Portals/0/Documents/evidence/clinical-handbooks/community-acquired-pneumonia-140227-en.pdf

17. Mandell LA, Wunderink RG, Anzueto A, et al; Infectious Diseases Society of America; American Thoracic Society. Infectious Diseases Society of America/American Thoracic Society consensus guidelines on the management of community-acquired pneumonia in adults. Clin Infect Dis. 2007;44(suppl 2):S27-S72. Medline:17278083 doi:10.1086/511159

18. tackling antimicrobial resistance and antimicrobial use: a pan-Canadian framework for action. September 5, 2017. Government of Canada. Accessed January 30, 2024.

© 2026 Tang B et al. *JAMA Network Open*.

https://www.canada.ca/en/health-canada/services/publications/drugs-health-products/tackling-antimicrobial-resistance-use-pan-canadian-framework-action.html

19. Ontario Agency for Health Protection and Promotion. Antimicrobial stewardship strategy: checklists. Public Health Ontario. 2016. Accessed January 30, 2024. https://www.publichealthontario.ca/apps/asp-strategies/data/pdf/ASP_Strategy_Checklists.pdf

20. Siddiqui S, Razzak J. Early versus late pre-intensive care unit admission broad spectrum antibiotics for severe sepsis in adults. Cochrane Database Syst Rev. 2010;2010(10):CD007081. Medline:20927754 doi:10.1002/14651858.CD007081.pub2

21. Overuse of tests and treatments in Canada. Canadian Institute for Health Information. 2022. Accessed January 30, 2024. https://www.cihi.ca/en/overuse-of-tests-and-treatments-in-canada

22. Rao VM, Levin DC. The overuse of diagnostic imaging and the Choosing Wisely initiative. Ann Intern Med. 2012;157(8):574-576. Medline:22928172 doi:10.7326/0003-4819-157-8-201210160-00535

23. Maberry M, Smith C, Go R. Clinical utility of monoclonal gammopathy screening in the evaluation of anemia. Blood. 2023;142(suppl 1):215. doi:10.1182/blood-2023-185125

24. Chami N, Simons JE, Sweetman A, Don-Wauchope AC. Rates of inappropriate laboratory test utilization in Ontario. Clin Biochem. 2017;50(15):822-827. Medline:28483406 doi:10.1016/j.clinbiochem.2017.05.004

25. Canadian Society of Internal Medicine. Eleven Tests and Treatments to Question. Choosing Wisely Canada; 2022.

26. Austin P. Using the standardized difference to compare the prevalence of a binary variable between two groups in observational research. Commun Stat Simul Comput. 2009;38:1228-1234. doi:10.1080/03610910902859574

© 2026 Tang B et al. *JAMA Network Open*.
